# Feasibility of a Pilot Crowdsourced Syndromic and Virological Surveillance Platform for Respiratory Illness in South Africa, CoughWatchSA, 2022

**DOI:** 10.1111/irv.70225

**Published:** 2026-02-09

**Authors:** Mvuyo Makhasi, Jocelyn Moyes, Daniela Paolotti, Mignon du Plessis, Fahima Moosa, Nicole Wolter, Phiwokuhle Ntombela, Siyabonga Mazibuko, Noluthando Duma, Jackie Kleynhans, Anne von Gottberg, Stefano Tempia, Sibongile Walaza, Cheryl Cohen

**Affiliations:** ^1^ Centre for Respiratory Diseases and Meningitis National Institute for Communicable Diseases, a Division of the National Health Laboratory Service Johannesburg South Africa; ^2^ School of Public Health University of the Witwatersrand Johannesburg South Africa; ^3^ Institute for Scientific Interchange Foundation Turin Italy; ^4^ School of Pathology, Faculty of Health Sciences University of the Witwatersrand Johannesburg South Africa

**Keywords:** CoughWatchSA, COVID19, DPS, feasibility, ILI, participatory surveillance, self‐swabbing

## Abstract

**Background:**

Digital participatory surveillance (DPS) may provide information on reported influenza‐like illnesses (ILI). Combining DPS with laboratory testing allows pathogen identification. We assessed the feasibility of DPS and home‐based self‐swabbing (HBSS) in South Africa.

**Methods:**

We enrolled a cohort of individuals aged ≥ 18 years who completed weekly respiratory symptoms questionnaires from March to October 2022. We calculated the weekly percentage of reported ILI and COVID‐19 and compared it with weekly private sector respiratory consultations (WPSRC). Symptomatic participants were offered HBSS for influenza and SARS‐CoV‐2 detection by polymerase chain reaction (PCR). We assessed six feasibility indicators.

**Results:**

Recruitment capability: Twenty‐six percent (249/954) of participants accessed the platform and enrolled, and 92% (81/88) of participants eligible for HBSS were enrolled. Acceptability: Fifteen percent (32/249) completed the acceptability questionnaire with 100% (32/32) willing to participate in future studies, and 16% (39/249) withdrew from the study. Representativeness: Fifty percent (125/249) were aged 18–39 years, predominantly female 71%, and 79% had a tertiary qualification. Reliability: Thirty‐eight percent (80/210) were active participants, median weekly active participation of 23% (interquartile range [IQR]: 19%–29%). Accuracy: Two percent (31/1440) and 25% (359/1440) of reports met ILI and COVID‐19, respectively. Influenza and SARS‐CoV‐2 were detected in 7% (6/81) and 32% (26/81) of tested samples, respectively. There was low correlation with WPSRC (0.08, 95% CI, 0.27–0.43) for ILI and (0.36, 95% CI, 0.11–0.62) for COVID19. Usefulness: Symptoms were reported in 32% (459/1440) of reports, and 11% (49/459) sought medical care.

**Conclusion:**

The study was feasible; however, low enrolment numbers limit power. Linkage to HBSS was successful and demonstrates the potential for pathogen confirmation.

## 1 Introduction

Facility‐based surveillance for respiratory illness is dependent on healthcare‐seeking practices, driven by the severity of illness and resource availability [1]. This impacts completeness and timeliness of reporting, which may lead to an underestimation of cases and the community burden of respiratory illness [1, 2, 3]. In low‐ and middle‐income countries (LMIC), access to medical care is not uniformly distributed compared with high‐income countries [4]. Innovative approaches to respiratory illness surveillance, which does not require individuals to seek care at healthcare facilities, may be more reliable for estimating health‐seeking behaviour and complement facility‐based surveillance [5].

Digital participatory surveillance (DPS) is a form of crowdsourced health data that utilizes mobile technology and/or the internet to allow community members to self‐report symptoms (or lack thereof) of respiratory illness [5, 6, 7, 8]. This allows public health officials to obtain data about respiratory illness directly from the community, including any healthcare seeking related to respiratory illness episodes [4]. DPS platforms provide real‐time data on reported respiratory illness episodes and healthcare‐seeking behaviour [9]. DPS platforms have also been used for animal disease surveillance and as a contribution towards One Health Disease Surveillance, such as AfyaData in Tanzania [10].

These platforms have been deployed in Europe, the United States and Australia and have been shown to provide estimates of proportion of medically to non‐medically attended illness [9, 11, 12]. DPS platforms have been implemented as pilot studies in LMIC settings such as Tanzania, Thailand and Cambodia for malaria [13] and a telephone‐based participatory surveillance platform for COVID‐19 in Lesotho [14]. However, there are few data on the implementation and acceptability of DPS in Africa for influenza‐like illness (ILI) surveillance.

Combining DPS with laboratory confirmation of infection has the advantage of confirming a respiratory viral infection, which may be useful in the scenario of an outbreak [15]. Home‐based self‐swabbing, for illness surveillance, has been implemented in settings such as Hong Kong [14], Germany [16], United Kingdom [17] and South Africa [18]. This has the potential for real‐time detection of circulating respiratory pathogens [15].

We aimed to assess the feasibility of a pilot DPS platform, CoughWatchSA, in South Africa with linkage to home‐based self‐swabbing (CoughCheck) using six indicators for feasibility: recruitment capability, acceptability, representativeness, reliability, accuracy and usefulness [19].

## 2 Materials and Methods

### 2.1 Study Population

CoughWatchSA was a prospective cohort of individuals from all nine provinces. Participation was voluntary and open to all consenting individuals aged ≥ 18 years who were willing to report respiratory symptoms (or lack thereof) weekly throughout the influenza season from March to October 2022. Participants who reported symptoms and lived in Johannesburg (Gauteng Province), Durban (KwaZulu‐Natal Province) and Cape Town (Western Cape Province) were considered eligible to enrol into CoughCheck for laboratory confirmation of influenza and SARS‐CoV‐2 infection [18].

### 2.2 Participant Enrolment and Retention

Participants were recruited through the National Institute for Communicable Diseases (NICD) website and social media platforms (*LinkedIn*, *Facebook* and *X)*, including radio and television interviews. Media resources, including video tutorials and social media messaging on the importance of DPS, were created and used to retain participants. The platform was data‐free through reverse‐billing to ensure participants did not incur any mobile data expenses. Participants could withdraw from the study at any point, and those who did were asked to complete a short questionnaire to determine their reasons for withdrawal.

### 2.3 Data Collection

#### 2.3.1 Pre‐Deployment Survey

A survey was published prior to the start of the pilot, in September 2020, through the NICD's social media platforms. This aim was to assess anticipated acceptability for a DPS platform and platform development before the pilot. The survey also aimed to ascertain platform preferences and willingness to report symptoms data on a weekly basis.

#### 2.3.2 CoughWatchSA Pilot

The main study was deployed between March and October 2022. There were three questionnaires completed:

1. An intake questionnaire completed once at enrolment included basic demographic information, lifestyle information and vaccination history for seasonal influenza and COVID‐19.
2. A symptoms questionnaire completed weekly for the duration of the pilot study included reported respiratory illness symptoms (or lack thereof), date of illness onset and healthcare‐seeking.
3. An acceptability questionnaire was completed at the conclusion of the pilot study to evaluate the acceptability, using the Theoretical Framework for Acceptability [20] described in Section 2.4.

#### 2.3.3 CoughCheck

Home‐based self‐swabbing allowed for sample collection from eligible participants (those who were symptomatic and resided in suburbs where testing was available). The detailed methods for sample collection and testing are published [18]; briefly, participants provided a self‐administered nasal sample from their residence or workplace. Sample collection materials were delivered to the participant following symptom reporting, and then samples were transported to NICD on ice for testing using the Allplex SARS‐CoV‐2/FluA/FluB/RSV polymerase chain reaction (PCR) assay (Seegene, Seoul, South Korea). Samples testing positive for influenza were further subtyped.

#### 2.3.4 Case Definitions

Participants were asked to report on a range of respiratory and systemic symptoms (fever, chills, runny nose, sneezing, sore throat, cough, shortness of breath, headache, muscle/joint pain, chest pain, fatigue, watery eyes, nausea, vomiting, diarrhoea, stomach ache, loss of smell, loss of taste). Adapted WHO case definitions for influenza‐like illness (ILI) and COVID‐19 were applied to the combination of reported symptoms. ILI was defined as reported fever or measured temperature ≥ 38°C and cough with symptom duration ≤ 10 days [21]. COVID‐19 was defined as duration of symptoms ≤ 10 days, with three or more of the following: fever, cough, fatigue, headache, myalgia, sore throat, coryza, shortness of breath, nausea and diarrhoea [22].

### 2.4 Data Analysis

Descriptive statistics were used to describe the epidemiological characteristics of participants, including demographics and reported symptoms, by means, medians and proportions as appropriate. All the analyses were done using *STATA 18 Standard Edition*. The six indicators for feasibility that were assessed are described below.

#### 2.4.1 Recruitment Capability

The proportion of participants who accessed the platform and enrolled was determined. Those who were eligible for CoughCheck and were successfully enrolled were calculated to assess how successful the linkage to home‐based self‐swabbing was. Enrolled participants were defined as participants who submitted at least one symptoms questionnaire and did not withdraw from the study.

#### 2.4.2 Acceptability

##### 2.4.2.1 Anticipated Acceptability

Responses to the pre‐deployment survey were assessed overall and by gender. This includes a willingness to report health data every week and platform preferences.

##### 2.4.2.2 Experienced Acceptability

This analysis was conducted in two parts (1) using the Theoretical Framework for Acceptability (TFA) described below and (2) participants' preferences on communications campaigns and recruitment strategies. Through TFA developed by Sekho et al. [20], acceptability was assessed across seven component constructs, namely: affective attitude (how an individual feels about the intervention); burden (the perceived amount of effort that is required to participate in the intervention); ethicality (the extent to which the intervention has good fit with an individual's value); intervention coherence (the extent to which the participant understands the intervention, how it addresses their condition and how it works); opportunity costs (the extent to which benefits, profits or values that must be given up to engage in the intervention); perceived effectiveness (the extent to which the intervention is perceived as likely to achieve its purpose); self‐efficacy (the participant's confidence that they can perform the behaviour(s) required to participate in the intervention) [20].

A 5‐point Likert scale was applied with scores ranging from 1 to 5 for *strongly disagree* to *strongly agree* [20]. The mean value and 95% confidence interval for each item or question were calculated, and a reliability coefficient (α) was calculated as a measure of internal consistency to determine overall acceptability, that is, α < 0.5 represents low acceptability and α ≥ 0.5 high acceptability.

For recruitment strategies and communications campaigns, responses to the acceptability questionnaire were analysed stratified by two age groups, 18–39 years and ≥ 40 years, to assess any differences across age groups using the Chi squared test.

The proportion of participants who withdrew from the study and their reason for withdrawal were reported.

#### 2.4.3 Representativeness

The characteristics of enrolled participants who withdrew from the study were compared to those who remained enrolled using the chi‐squared test. The following individual characteristics were compared: gender, province, education level, employment status, seasonal influenza and COVID‐19 vaccination histories. The percentage of individuals in the study population was compared to the national census data published in 2022 [23] by age group and education level. Comparison by sex could not be done because we collected gender identity instead of sex as a biological characteristic and we cannot infer sex from gender identity.

#### 2.4.4 Reliability

Active participants are defined as those who submitted at least two symptoms questionnaires until end of follow‐up. The weekly percentage of active participants was calculated as: the total number of active participants submitting symptoms questionnaires in the reporting week over the total number of enrolled participants. The median weekly percentage of participants who submitted a report was also determined to assess the reliability of reported data.

#### 2.4.5 Accuracy

Active participants were included in the analysis of correlation of timing of illness with timing of respiratory illness consultations, following a similar approach to a previous DPS study in Italy [24]. The weekly percentage of cases meeting either ILI or COVID‐19 case definitions was determined by the total number of cases fitting the ILI or COVID‐19 case definition over the total number of submitted symptom questionnaires in the reporting week.

Weekly private sector respiratory consultation rates as a proportion of total consultations were obtained from a private healthcare provider database [25]. Percentage of respiratory consultations as a proportion of all consultations is not available from sentinel surveillance programmes as denominator data are not routinely collected. The percentage of patients consulting for respiratory illness as a proportion of all consultations was calculated. For ILI, we used the number of ICD‐10 codes reported as J10‐J18 (for suspected pneumonia‐ and influenza‐related consultations) and for COVID‐19, we used the ICD‐10 codes U07.1 and U07.2. This was aggregated weekly and a moving average with a window size of 3 weeks was used to smooth the time series for both ILI and COVID‐19. The weekly private sector respiratory consultation data were compared with the weekly percentage of cases of ILI or COVID‐19 from our study using cross‐correlation with a 1‐week lag. The correlation coefficient (*r*) is a range of −1 to 1, where values close to zero are considered poor or no correlation and values > 0.5 are considered moderate to good correlation.

The detection rates for influenza and SARS‐CoV‐2 (for those enrolled in CoughCheck) were calculated among all individuals with laboratory results as the total number testing PCR positive over the total number of individuals tested. We also determined the percentage of participants who tested positive for influenza and SARS‐CoV‐2 that met the respective case definitions.

#### 2.4.6 Usefulness

The percentage of reports that had symptoms and among those that sought medical care was calculated and compared by syndrome (ILI and COVID‐19).

#### 2.4.7 Ethics

Interested individuals, using an online survey‐based platform (Real‐time Electronic Data Capture, REDCap), were requested to read a participant information sheet and provide electronic informed consent for enrolment. Ethical approval was obtained from the University of the Witwatersrand Human Research Ethics Committee, reference: M2008135.

## 3 Results

### 3.1 Recruitment Capability

**FIGURE 1:**
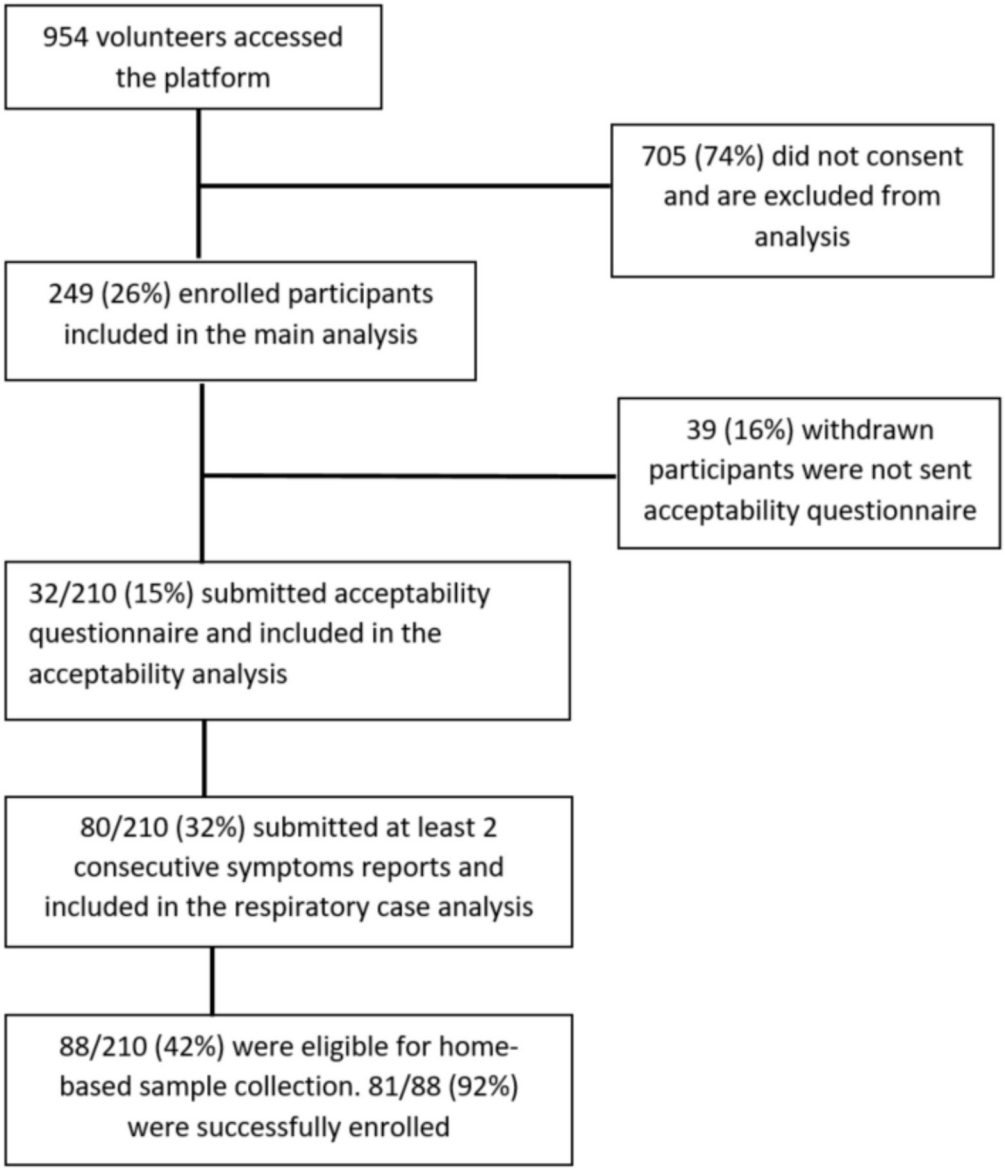
Flow diagram of participants in the CoughWatchSA pilot study, South Africa, 2022.

A total of 954 individuals accessed the platform and 26% (249/954) enrolled in the study (Figure 1). Of the enrolled participants, 35% (88/249) were eligible for home‐based self‐swabbing and 92% (81/88) were successfully enrolled.

### 3.2 Acceptability

#### 3.2.1 Anticipated Acceptability

Of the 1001 volunteers who completed the pre‐deployment survey in September 2020, 98% (977/1001) were included in the final analysis, where four individuals had missing gender or incomplete questionnaires. The majority of volunteers were in the 31‐ to 59‐year age group (71%, 698/977); 60% (586/977) were female; 51% (496/977) lived in Gauteng Province; 93% (909/977) lived in urban areas; 88% (856/977) indicated a willingness to report symptoms regularly on an online application; 92% (900/977) had consistent internet access; and 35% (337/977) preferred WhatsApp for the platform. There were no differences between male and female respondents (Table 1).

**TABLE 1:** Individual characteristics of participants who completed the user anticipated acceptability questionnaire, that is, pre‐deployment survey, September 2020, South Africa.

Characteristic	Overall (*N* = 977) *n* (%)	Female (*N* = 586) *n* (%)	Male (*N* = 391) *n* (%)	*p*
Age category				0.094
18–30 years	224 (24)	122 (21)	102 (26)	
31–59 years	698 (70)	429 (73)	261 (67)	
60+ years	63 (6)	35 (6)	28 (7)	
Province				0.278
Eastern Cape	49 (5)	28 (5)	21 (5)	
Free State	34 (4)	19 (3)	15 (4)	
Gauteng	496 (51)	310 (53)	186 (48)	
KwaZulu‐Natal	116 (12)	72 (12)	44 (11)	
Limpopo	16 (2)	11 (2)	5 (1)	
Mpumalanga	29 (3)	15 (3)	14 (4)	
North West	33 (3)	21 (4)	12 (3)	
Northern Cape	20 (2)	7 (1)	13 (3)	
Western Cape	182 (19)	102 (17)	80 (21)	
Type of residential area				0.634
Urban	909 (93)	543 (93)	366 (94)	
Rural	68 (7)	42 (7)	25 (6)	
Willingness to report symptoms				0.095
Yes	856 (88)	505 (86)	351 (90)	
Home language preference				0.540
Yes	685 (70)	415 (71)	270 (69)	
Access to internet				0.376
Yes	900 (92)	544 (93)	356 (91)	
Preferred platform				0.200
Facebook	123 (13)	85 (15)	38 (10)	
Mobile app	312 (32)	185 (32)	127 (33)	
Web survey	161 (15)	90 (15)	71 (18)	
WhatsApp	337 (35)	202 (35)	134 (34)	
None	44 (5)	24 (4)	20 (5)	

#### 3.2.2 Experienced Acceptability

Only 15% of participants (32/210) completed the acceptability questionnaire after enrolling in the main study. Through the TFA component constructs, the overall acceptability using the reliability coefficient (α) was 0.8412 an indicator for high acceptability (Table 2). There were no differences between younger (18–39 years) and older (≥ 40 years) age groups when comparing the communication and recruitment strategies responses (Table 3). Of the 32 participants who completed the acceptability questionnaire, 82% (26/32) heard about the study from the NICD digital platforms; 88% (28/32) indicated they were interested in the home‐based self‐swabbing study; 94% (30/32) indicated they would still participate if the home‐based testing was not available and all participants indicated their willingness to participate in future studies.

**TABLE 2:** Experienced acceptability using the Theoretical Framework of Acceptability in CoughWatchSA, South Africa 2022 (*N* = 32).

Variable	Mean score/5 (95% CI)	α^a^ (reliability coefficient)
Experienced acceptability		0.8412
Affective attitude	4.22 (3.96–4.47)	
Burden	4.41 (4.13–4.68)	
Ethicality	4.53 (4.29–4.77)	
Opportunity costs	4.69 (4.52–4.86)	
Perceived effectiveness	3.97 (3.66–4.28)	
Self‐efficacy	4.44 (4.20–4.68)	
Intervention coherence	4.53 (4.33–4.74)	

^a^ Reliability coefficient assess level of experienced acceptability overall from the seven component constructs for acceptability where α ranges between 0 and 1; α < 0.5 means low acceptability and α ≥ 0.5 means high acceptability.

**TABLE 3:** Preferred communications strategies for participant acquisition and enrolment, from CoughWatchSA, South Africa, 2022.

Variable	Total *N* = 32 (%)	18–39 years *N* = 8 (%)	40+ years *N* = 24 (%)	*p*
How did you first hear about CoughWatchSA?				0.148
NICD^a^ digital platforms	26 (82)	5 (63)	21 (88)	
Press release, newspaper, online publication	3 (9)	1 (13)	2 (8)	
Other	3 (10)	2 (25)	1 (4)	
Interested in home‐based testing				0.705
Yes	28 (88)	7 (88)	21 (88)	
Would you participate if home‐based testing not available				0.444
Yes	30 (94)	7 (88)	23 (96)	
Did free data service incentivize your participation				0.423
Yes	7 (22)	1 (13)	6 (25)	
Motivation for participation				0.250
Contribution to respiratory disease surveillance	31 (97)	7 (88)	24 (100)	
Access to home‐based testing	1 (3)	1 (13)	0 (0)	
Willingness to enrol in future studies				—
Yes	32 (100)	8 (100)	24 (100)	

^a^ National Institute for Communicable Diseases.

A total of 16% (39/249) participants withdrew after completing one symptoms questionnaire. Among this group, the primary reason was lack of time to participate (72%, 23/32), followed by an overwhelming number of email notifications from the study (31%, 10/32).

### 3.3 Representativeness

There were no differences in individual characteristics between enrolled and withdrawn participants (Table 4). Just over a third of enrolled participants were aged 30–39 years (37%, 93/249), most participants were women (71%, 178/249), a majority had some tertiary qualification (79%, 196/249) and 84% (210/249) were employed or earning an income. A quarter of participants, 25% (62/249), reported receiving the annual seasonal influenza vaccine in 2022, and 85% (212/249) reported receiving at least one COVID‐19 vaccine dose. Most enrolments were in Gauteng (44%, 109/249), followed by the Western Cape (27%, 66/249) and KwaZulu‐Natal (14%, 35/249) (Figure S1). Comparing the demographics of our cohort to the 2022 national census data, our cohort differs by age group: 13% of 18–29 years old versus 21% in census data; 37% of 30–39 years old versus 17% in census data; and 26% of 40–49 years old versus 12% in census data; the differences were statistically significant (*p* < 0.001) (Table S1). Furthermore, our study population differs by education level: 79% with tertiary qualification versus 12% in census data and 4% with no qualification versus 50% in census data.

**TABLE 4:** Characteristics of enrolled and withdrawn participants in the CoughWatchSA study, South Africa, 2022.

Characteristic	Total *n* (%)	Enrolled participants^a^ *n* (%)	Withdrawn participants *n* (%)	*p*
	*N* = 249	*N* = 210	*N* = 39	
Age group (years)				0.825
18–29	32 (13)	27 (13)	5 (13)	
30–39	93 (37)	79 (38)	14 (36)	
40–49	65 (26)	53 (25)	12 (31)	
50–59	32 (13)	29 (14)	3 (8)	
≥ 60	27 (11)	22 (11)	5 (13)	
Gender				0.162
Male	71 (29)	58 (28)	13 (34)	
Female	177 (71)	153 (72)	24 (66)	
Prefer not to say	1 (0)	0 (0)	1 (0)	
Prefer to self‐describe	0 (0)	0 (0)	0 (0)	
Highest education level				0.548
Tertiary qualification^b^	196 (79)	166 (79)	30 (77)	
Matric certificate	42 (17)	36 (17)	6 (15)	
No qualification^c^	11 (4)	8 (4)	3 (8)	
Employment status				0.411
Employed/earning income	210 (84)	178 (85)	32 (82)	
Unemployed	39 (16)	32 (15)	7 (18)	
Daily transport mode				0.193
Private	15 (6)	11 (5)	4 (10)	
Public	234 (94)	199 (95)	35 (90)	
Number of household members				0.081
< 5	225 (90)	187 (89)	38 (97)	
≥ 5	24 (10)	23 (11)	1 (3)	
Seasonal influenza vaccine				0.188
Vaccinated	62 (25)	55 (26)	7 (18)	
Not vaccinated	187 (75)	155 (74)	32 (82)	
Received at least one dose of COVID‐19 vaccine				0.542
Yes	212 (85)	179 (85)	33 (85)	
No	37 (15)	31 (15)	6 (15)	

^a^ Enrolled participants are those who submitted at least one symptoms questionnaire and did not withdraw from the study.

^b^ Tertiary qualification includes higher certificate, undergraduate degree or diploma, post graduate qualification.

^c^ No qualification means they may have attended some primary or secondary school but did not complete and have no other qualifications.

### 3.4 Reliability

**FIGURE 2:**
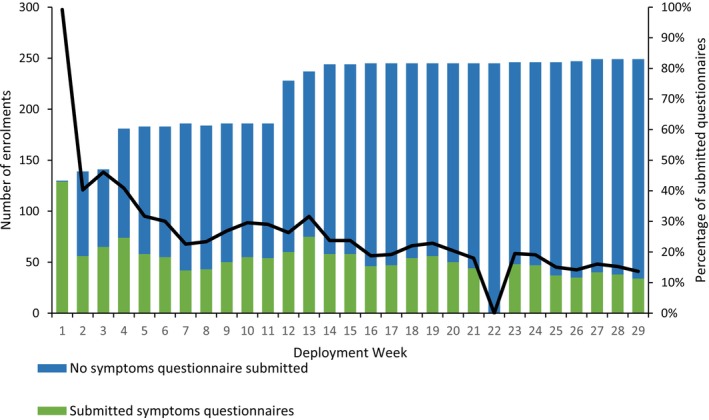
Total number of enrolments and weekly percentage of active participants from CoughWatchSA, South Africa, 2022. *There were no questionnaires submitted in Week 22 due to the network server being down.

Thirty‐eight percent (80/210) of participants submitted more than one weekly symptoms questionnaire (active participants), where the median weekly percentage of active participants was 23% (interquartile range [IQR]: 19%–29%) with the highest recorded in Week 3 (45%, 74/165). Participation fluctuated weekly with 54 enrolled participants in Week 1 and peaking at 249 enrolled participants in Week 23 (Figure 2). Data were not submitted for Week 22 due to the application server being down.

### 3.5 Accuracy

**FIGURE 3:**
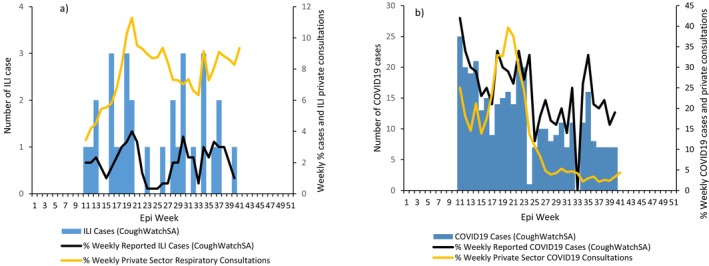
Weekly number and percentage of individuals reporting symptoms that met (a) influenza‐like‐illness and (b) COVID‐19 case definitions from CoughWatchSA, South Africa, 2022, compared to respiratory illness consultations from private healthcare data.

A total of 1440 symptoms questionnaires were submitted over the 29 weeks from active participants. Of these, 2% (31/1440) met the ILI and 25% (359/1440) met the COVID‐19 case definitions, respectively (Figure 3). There was no correlation between the weekly percent of ILI and COVID‐19 cases with respiratory illness private consultations, correlation coefficient (*r*) = 0.08 (95% confidence interval [CI], −0.27 to 0.43) and (*r*) = 0.36 (95% CI, 0.11–0.62), respectively. Overall, 7% (6/81) of tested individuals tested positive for influenza and 32% (26/81) for SARS‐CoV‐2 (Table S2). Among the influenza‐positive cases, 50% (3/6) met the ILI case definition, and 77% (20/26) among SARS‐CoV‐2 cases met the COVID‐19 case definition.

### 3.6 Usefulness

Of the 1444 symptom reports, 32% (459/1440) reported symptoms and all reported ≥ 1 symptom, of these 11% (49/459) sought medical care. Among ILI cases (*n* = 31), 58% (18/31) sought medical care, and 26% (43/359) of COVID19 cases sought medical care.

## 4 Discussion

Our results provide important insights into the feasibility and potential use of DPS for respiratory illness in South Africa. We found the study to be feasible across all indicators; however, accuracy in determining the timing of the respiratory season was poor. We were able to identify reported cases fitting ILI and COVID‐19 case definitions. The linkage of eligible participants to home‐based testing was successful, resulting in the detection of influenza and SARS‐CoV‐2 viruses. The combination of DPS and home‐based self‐swabbing is a novel contribution from the study and shows great promise for close to real‐time detection of circulating pathogens directly in the community.

A scoping review of 18 participatory surveillance systems globally showed that recruitment capability, which includes participant acquisition and retention, remains the biggest challenge for DPS [26], similar to our experience. Despite various communications campaigns on social media, television and radio interviews, we could not increase the number of enrolments beyond 249 participants. In Week 11, we presented our study to a forum that includes provincial epidemiologists, the National Department of Health and communication specialists who shared the study through their platforms, resulting in an increase in enrolments. However, participation waned over time due to reporting fatigue and reaching end of season, consistent with findings from [24].

We found a high anticipated acceptability from the pre‐deployment survey, and the experienced acceptability was high among the small number of participants who responded. The majority of participants are a public health audience shown by the majority of participants who learned about the study through NICD platforms, which is consistent with findings from other settings such as Italy [3], the United States [4] and Australia [12]. We found a similar percentage of participants who withdrew from our study and those who completed the acceptability questionnaire. This suggests that more evidence is required for prove acceptability of DPS in our setting in the next phase of this study. However, The Theoretical Framework for Acceptability (TFA) developed by Sekho et al. [20] provides a quantitative metric for measuring acceptability and our study contributes to this body of work.

Overall, our cohort was mostly educated, middle‐aged female participants, which is consistent with findings from [5, 11, 27]. Our study population is not representative of the broader population of South Africa. However, DPS data have previously been shown to complement facility‐based surveillance by providing data on age‐groups that are not well represented in facility‐based surveillance and improved seasonal influenza vaccination coverage from a population that is not commonly surveyed [24]. Our study demonstrates this by showing a higher seasonal influenza and COVID‐19 vaccination coverage as compared to Viral Watch, a sentinel surveillance programme for ILI in South Africa that reported 12% seasonal influenza vaccine coverage in 2022 [28]; CoughCheck, a home‐based self‐swabbing study, reported 11% seasonal influenza vaccine coverage in 2022 [29], compared to 25% in our study. The majority of our study population (70%) were NICD staff who have convenient and free access to the seasonal influenza vaccine, compared to the population in CoughCheck, which was a more heterogeneous group. This may account for the difference in influenza vaccine coverage.

Although the majority of participants indicated a willingness to report symptom information in future deployments, our study shows that participant retention is important to improve reliability of the data. We had no data on participants who accessed the platform but did not enrol, and this may have provided valuable insights into participant acquisition and retention. More participants preferred a WhatsApp or mobile application from the pre‐deployment survey. This may indicate a need for a flexible mobile platform and this is unique to our setting as other settings implement web‐based applications [9].

In our study, we observed that 2% of reports fitted the ILI case definition. This compares to 1%–2% reported in Flu Near Year in the United States for 2015–2016 to 2018–2019 influenza seasons [4]; 1%–6% of ILI in Flu Tracking in 2020 [30]. In InfluenzaNet, weekly incidences were calculated per 100,000 population, an analysis we did not conduct making it difficult to compare to our data [9].

We were unable to demonstrate correlation with private sector respiratory consultations, despite using time series data with a week lag. Published literature shows a good correlation between reported illness and private consultations as demonstrated in Italy and France [24, 31] and a scoping review on 10 years of internet‐based surveillance systems [26]. Furthermore, our study excluded children below 18 years old, excluding a large group of individuals impacted by respiratory illness. This may have affected the accuracy of the reported cases in our study.

The link to home‐based self‐swabbing, although limited to certain areas, was successful and provides evidence for the feasibility of close to real‐time confirmation of circulating respiratory pathogens through DPS. This demonstrates the flexibility of DPS to link participants to laboratory confirmations and the potential use of this platform to provide seasonal influenza vaccine effectiveness (VE) [29].

The percentage of participants who were symptomatic and sought medical care was lower than that found in the PHIRST Study in South Africa, a prospective community observational cohort study [32], where 25% of individuals with symptoms sought medical care compared to 11% in our study. Health‐seeking data among *Flu Near You* participants, a DPS platform in the United States [4], showed healthcare seeking for ILI ranged from 22% to 36% between 2015–2019 influenza seasons. This difference could be attributed to the low enrolment numbers and inconsistent participation.

There are a number of limitations in our study. First, we had a low number of enrolments and our non‐response rate increased over time leading to participation bias. This affected our correlation analysis with private sector respiratory consultations and negatively impacted the accuracy indicator for feasibility. Our results may not be generalizable to rural and low‐income areas and our study population is not representative of the general population of South Africa. Our study excluded children ≤ 18 years old, particularly those < 5 years, which are a very important group for respiratory illness. Due to the low response rate for the acceptability questionnaire, bias may have been introduced in this select group of participants and may suggest that this study was not acceptable to a large group in the study.

The following should be considered in the next phase of the study: Recruitment capability can be improved by implementing strategies such as the use of political ambassadors or health influencers to solicit participation as done in Australia [12]; financial incentives to encourage participation as in Thailand [13]; and having a consistently deployed DPS platform for an extended period of time where a loyal cohort has been established such as in InfluenzaNet [9]. The use of a WhatsApp mobile application or Unstructured Supplementary Service Data (USSD) as a point of access may be a useful addition to the web based platform to improve participant retention. Overall, improving enrolment numbers, participation rates and considering adding a younger age group may improve the correlation between DPS data and private sector respiratory consultation rates.

## 5 Conclusions

This pilot study has provided important insights on the feasibility of DPS for identifying respiratory symptoms and cases of ILI and COVID‐19 and the linkage to real‐time laboratory testing for influenza and SARS‐CoV‐2. These findings will inform the next DPS deployment in South Africa to improve feasibility and possibly assist other countries similar to our context in the planning, design, development and refinement of a DPS platform.

## Author Contributions

**Mvuyo Makhasi:** conceptualization, data curation, formal analysis, investigation, methodology, project administration, software, validation, visualization, writing – original draft, writing – review and editing. **Jocelyn Moyes:** conceptualization, formal analysis, investigation, methodology, project administration, validation, writing – review and editing. **Daniela Paolotti:** conceptualization, formal analysis, investigation, validation, supervision, writing – review and editing. **Mignon du Plessis:** conceptualization, methodology, project administration, writing – review and editing. **Fahima Moosa:** conceptualization, data curation, methodology, project administration, writing – review and editing. **Nicole Wolter:** conceptualization, methodology, validation, writing – review and editing. **Phiwokuhle Ntombela:** conceptualization, data curation, methodology, project administration, software, writing – review and editing. **Siyabonga Mazibuko:** data curation, methodology, project administration, software, writing – review and editing. **Noluthando Duma:** conceptualization, data curation, methodology, project administration, writing – review and editing. **Jackie Kleynhans:** conceptualization, methodology, validation, writing – review and editing. **Anne von Gottberg:** conceptualization, investigation, methodology, validation, writing – review and editing. **Stefano Tempia:** conceptualization, formal analysis, investigation, methodology, supervision, validation, writing – review and editing. **Sibongile Walaza:** conceptualization, formal analysis, investigation, methodology, project administration, supervision, validation, writing – review and editing. **Cheryl Cohen:** conceptualization, formal analysis, investigation, methodology, project administration, supervision, validation, writing – review and editing.

## Funding

This study was supported by Sanofi.

## Conflicts of Interest

C.C. has received grant funds from the US Centers for Disease Control and Prevention, the Gates Foundation, the Taskforce for Global Health and Sanofi Pasteur. S.W. has received grant funds from the US Centers for Disease Control and Prevention, the Gates Foundation and the Taskforce for Global Health. J.M. has received grant funding from Sanofi Pasteur. N.W. has received grant funding from the Gates Foundation and the US Centers for Disease Control and Prevention (US‐CDC). M.d.P. has received grant funding from the Gates Foundation and the US Centers for Disease Control and Prevention (US‐CDC). A.v.G. received grant funding for research from Centers for Diseases Control and Prevention (US‐CDC).

## Supporting information


**Figure S1:** Total number of enrolments by province in CoughWatchSA, South Africa, 2022 (GP = Gauteng Province; WC = Western Cape; KZN = KwaZulu Natal; MP = Mpumalanga Province; NW = North West; NC = Northern Cape; LP = Limpopo Province; EC = Eastern Cape; FS = Free State).
**Table S1:** Comparison of individual characteristics to National Census Data 2020 obtained from [20].
**Table S2:** Number of influenza or SARS‐CoV‐2 positive test results from CoughWatchSA participants who were successfully enrolled in the home‐based sample collection (N = 81), South Africa, March–October 2022.

## Data Availability

The data that support the findings of this study are available on request from the corresponding author. The data are not publicly available due to privacy or ethical restrictions.
