# Supplementary material for: Feasibility of a Pilot Crowdsourced Syndromic and Virological Surveillance Platform for Respiratory Illness in South Africa, CoughWatchSA, 2022
Source: Influenza Other Respir Viruses. 2026 Feb 9;20(2):e70225. doi: 10.1111/irv.70225 (PMC12886190; doi:10.1111/irv.70225)
Supplement: Supplementary file 1 — Figure S1: Total number of enrolments by province in CoughWatchSA, South Africa, 2022 (GP = Gauteng Province; WC = Western Cape; KZN = KwaZulu Natal; MP = Mpumalanga Province; NW = North West; NC = Northern Cape; LP = Limpopo Province; EC = Eastern Cape; FS = Free State). Table S1: Comparison of individual characteristics to National Census Data 2020 obtained from [20]. Table S2: Number of influenza or SARS‐CoV‐2 positive test results from CoughWatchSA participants who were successfully enrolled in the home‐based sample collection (N = 81), South Africa, March–October 2022. [file IRV-20-e70225-s001.docx]

**Feasibility of a Pilot Crowdsourced Syndromic and Virological Surveillance Platform for Respiratory Illness in South Africa, CoughWatchSA, 2022**

**Supplementary Material**

**Figures**

Figure S1: Total number of enrolments by province in CoughWatchSA, South Africa, 2022

(GP=Gauteng Province; WC=Western Cape; KZN=KwaZulu Natal; MP=Mpumalanga Province; NW=North West; NC=Northern Cape; LP=Limpopo Province; EC=Eastern Cape; FS=Free State)

Table S1: Comparison of individual characteristics to National Census Data 2020 obtained from [22]

| **Characteristic** | **CoughWatchSA**  **N=249**  **n (%)** | **National Census Data 2022**  **N=62,027,503^a^**  **n (%)** | **p-value** |
| --- | --- | --- | --- |
| **Age group (years)** |  |  | <0.001 |
| 18-29 | 32 (13) | 12,852,186 (21) |  |
| 30-39 | 93 (37) | 10,815,426 (17) |  |
| 40-49 | 65 (26) | 7,478,731 (12) |  |
| 50-59 | 32 (13) | 5,265,591 (8) |  |
| >=60 | 27 (11) | 6,102,723 (10) |  |
| **Education level** |  |  | <0.001 |
| Tertiary qualification^b^ | 196 (79) | 4,602,765/37,258,088^c^ (12) |  |
| Matric certificate | 42 (17) | 14,122,681/37,258,088^c^ (38) |  |
| No qualification | 11 (4) | 18,552,336/37,258,088^c^ (50) |  |

^a^ Population size from 2022 national census data, age bands 0-17 are exclude from the table but the denominator represents the entire population as reported in the 2022 National Census data

^b^ Tertiary qualification defined as any post school qualification

^c^ population denominator from National Census data accounting for individuals aged 20 years and older.

Table S2: Number of influenza or SARS-CoV-2 positive test results from CoughWatchSA participants who were successfully enrolled in the home-based sample collection (N=81), South Africa, March-October 2022.

| **Province** | **Influenza**  **n/N (%)** | **SARS-CoV-2** **n/N (%)** |
| --- | --- | --- |
| Johannesburg | 5/37(14) | 10/37 (27) |
| Durban | 0/25 (0) | 14/25 (56) |
| Cape Town | 1/19 (5) | 2/19 (11) |
| **Overall** | **6/81 (7)** | **26/81 (32)** |
